# Tracking Changes in Corticospinal Excitability During Visuomotor Paired Associative Stimulation to Predict Motor Resonance Rewriting

**DOI:** 10.3390/brainsci15030257

**Published:** 2025-02-27

**Authors:** Giacomo Guidali, Nadia Bolognini

**Affiliations:** 1 Department of Psychology and Milan Center for Neuroscience—NeuroMI, University of Milano-Bicocca, Piazza dell’Ateneo Nuovo 1, 20126 Milan, Italy; 2 Laboratory of Neuropsychology, IRCCS Istituto Auxologico Italiano, 20122 Milan, Italy

**Keywords:** motor resonance, action observation, paired associative stimulation, mirror neuron system, associative plasticity, corticospinal excitability, TMS

## Abstract

**Background/Objectives.** Mirror properties of the action observation network (AON) can be modulated through Hebbian-like associative plasticity using paired associative stimulation (PAS). We recently introduced a visuomotor protocol (mirror–PAS, m-PAS) that pairs transcranial magnetic stimulation (TMS) over the primary motor cortex (M1) with visual stimuli of ipsilateral (to TMS) movements, leading to atypical corticospinal excitability (CSE) facilitation (i.e., motor resonance) during PAS-conditioned action observation. While m-PAS aftereffects are robust, little is known about markers of associative plasticity during its administration and their predictive value for subsequent motor resonance rewriting. The present study aims to fill this gap by investigating CSE modulations during m-PAS and their relationship with the protocol’s aftereffects. **Methods.** We analyzed CSE dynamics in 81 healthy participants undergoing the m-PAS before and after passively observing left- or right-hand index finger movements. Here, typical and PAS-conditioned motor resonance was assessed with TMS over the right M1. We examined CSE changes during the m-PAS and used linear regression models to explore their relationship with motor resonance modulations. **Results.** m-PAS transiently reshaped both typical and PAS-induced motor resonance. Importantly, we found a gradual increase in CSE during m-PAS, which predicted the loss of typical motor resonance but not the emergence of atypical responses after the protocol’s administration. **Conclusions.** Our results suggest that the motor resonance reshaping induced by the m-PAS is not entirely predictable by CSE online modulations. Likely, this rewriting is the product of a large-scale reorganization of the AON rather than a phenomenon restricted to the PAS-stimulated motor cortex. This study underlines that monitoring CSE during non-invasive brain stimulation protocols could provide valuable insight into some but not all plastic outcomes.

## 1. Introduction

Associative sensorimotor learning plays a crucial role in shaping mirror properties of the human brain [1,2,3]. Recent studies highlight the feasibility of modulating mirror neuron responses using experimental paradigms leveraging this form of learning, e.g., [4,5,6,7,8,9] or the induction of Hebbian-like associative plasticity through non-invasive brain stimulation protocols [10,11,12,13,14,15,16].

Considering the domain of action observation, visuomotor properties of human mirror neurons can be studied non-invasively by exploiting the motor resonance phenomenon, i.e., the corticospinal excitability (CSE) enhancement detectable during the observation of biological movements [17,18,19]. This phenomenon is assessed by recording motor-evoked potentials (MEPs) from the transcranial magnetic stimulation (TMS) of the primary motor cortex (M1). It is thought to reflect the activation of mirror neuron populations located in the ventral premotor cortex (PMv), a key hub of the action observation network AON [20], which has a direct connection with M1, in turn influencing motor system excitability during action observation, e.g., [13,21,22,23,24,25,26].

Motor resonance responses can be experimentally modulated with paired associative stimulation (PAS), a class of non-invasive brain stimulation protocols repeatedly coupling a peripheral and a cortical stimulation activating the same cortical area/circuit, in turn inducing Hebbian associative plasticity in the target system; for reviews, see [27,28,29]. Our research group introduced a visuomotor version of the PAS, targeting the AON, called the mirror PAS, m-PAS [12], which reliably induces a reshaping of motor resonance responses for simple movements by leveraging their hemispheric lateralization, i.e., an increase in MEPs is detectable only when stimulation is applied to the M1 contralateral to the observed movement, e.g., [30]. During the m-PAS, TMS pulses are repeatedly delivered over M1 in conjunction with visual stimuli showing finger movements performed by the hand ipsilateral to the stimulation site, a condition that at baseline is unrelated to CSE facilitation. Following its administration, an atypical motor resonance response (i.e., not present in the baseline) emerges, as indexed by CSE facilitation in the ipsilateral hemisphere when observing the PAS-conditioned movement [12]. Interestingly, this emergence occurred at the cost of the typical response, significantly reduced after the protocol’s administration [16]. The m-PAS corticospinal effects are also accompanied by modulations of AON activation behavioral markers, i.e., automatic imitation [15] and TMS-evoked M1 functional connectivity in the alpha and beta bands during action observation [16]. Altogether, this evidence suggested that the (transient) motor resonance rewriting brought about by the protocol is the product of a large-scale reorganization of the AON not only limited to the stimulated M1 [12,15,16].

Recently, a cortico-cortical PAS targeting PMv-to-M1 connectivity was also found effective in modulating typical motor resonance and automatic imitation [13,14]. As said before, CSE facilitation during action observation is thought to reflect excitatory connections between ventral premotor regions of the AON and M1; for reviews, see [20,31], and this explains why targeting such a cortico-cortical connection could influence AON activation proxies [13,14]. In this vein, we can speculate that the m-PAS also recruits a premotor-to-motor pathway, even if not directly stimulated with TMS [12,16]. Notably, using the PMv-M1 cortico-cortical PAS, Turrini et al. (2022, 2023, 2024) found a gradual enhancement of MEP amplitude during its administration, suggesting that, at least for this ccPAS version, CSE could be used as a reliable online marker of Hebbian associative plasticity induction within M1. Still, this enhancement was not investigated in relation to the aftereffects of the protocol (i.e., whether CSE modulations during PAS are somehow predictive of the protocol’s outcomes at the single-subject level) [14,32,33].

Hence, assuming a shared neurophysiological substrate between the PMv-M1 cortico-cortical PAS and the m-PAS, CSE could also be modulated similarly during the latter visuomotor protocol. This could reflect the (gradual) induction of associative plasticity within the motor system and, likely, a plastic rewiring of the stimulated M1 inter-areal communication, responsible for the (atypical) AON recruitment at the sight of the PAS-conditioned movement after the protocol’s administration [16]. If CSE modulations during the m-PAS reflect the induction of state-dependent associative plasticity within the AON, their magnitude may predict the reshaping of motor resonance patterns. Importantly, research is still lacking on whether the online modulation of CSE can predict subsequent m-PAS-induced plasticity. This investigation could provide valuable information on the neurophysiological underpinnings of plastic modulations induced by the protocol and, in a broader perspective, on how associative plasticity within the AON develops during a visuomotor protocol based on (passive) action observation.

Given these premises, in the present work, we investigate possible CSE changes during m-PAS administration and whether they predict the protocol’s aftereffects on typical and experimentally induced motor resonance by aggregating data from previous studies conducted by our research group [12,15,16]. Driven by previous evidence on cortico-cortical PAS [14,32], we expected that an enhancement of M1 reactivity already occurred online during the administration of the m-PAS. If this is the case, we further hypothesize that its magnitude may be related to the motor resonance reshaping found after this visuomotor version of the PAS [12,15,16]. From a broader perspective, our investigation aims to shed better light on possible markers of sensorimotor associative plasticity induction during the administration of the m-PAS, exploring whether they could predict the magnitude of subsequent aftereffects.

## 2. Materials and Methods

### 2.1. Participants

We took datasets of the present work from 93 subjects who participated in a series of previous m-PAS studies conducted by our research group [12,15,16]. None of these studies had explored the possible modulation of CSE during the m-PAS protocol. For the present work, we considered only data from sessions where the m-PAS administered to the participant had the parameters found effective in all our studies (i.e., with an inter-stimulus interval—ISI—between paired stimulations of 25 ms, depicting right-hand index finger movements, and with TMS delivered over right M1). We excluded from our initial dataset participants whose MEPs were not recorded during the m-PAS protocol due to technical issues (*n* = 8). Four participants took part in more than one experiment; hence, we considered data only from the first experiment in which they participated. All this considered, the final sample included 81 right-handed healthy participants (35 males, mean age ± standard deviation—SD: 23.7 ± 2.9 years; mean education ± SD: 15.6 ± 1.9 years; mean Edinburgh handedness inventory score ± SD: 74 ± 15.9%). To assess whether this number of participants is sufficient to obtain reliable results from linear regression analyses, we ran an a priori power analysis with the software G*Power 3.1 [34]. The power analysis (*f*^2^ = 0.15—corresponding to a medium desired effect size [35], alpha error level: *p* = 0.05; statistical power = 0.9, actual power = 0.9) suggested at least 73 participants to achieve enough statistical power. All the original experiments were performed following the ethical standards of the Declaration of Helsinki. Before taking part in the study, participants gave their written informed consent. The present work obtained the approval of the Ethical Committee of the University of Milano-Bicocca on 24 July 2024 (protocol number 881-24). The study’s dataset and analysis are publicly available at Open Science Framework [OSF—https://osf.io/7gsut/ (accessed on 25 February 2025)].

### 2.2. m-PAS and Action Observation Task

**Figure 1:**
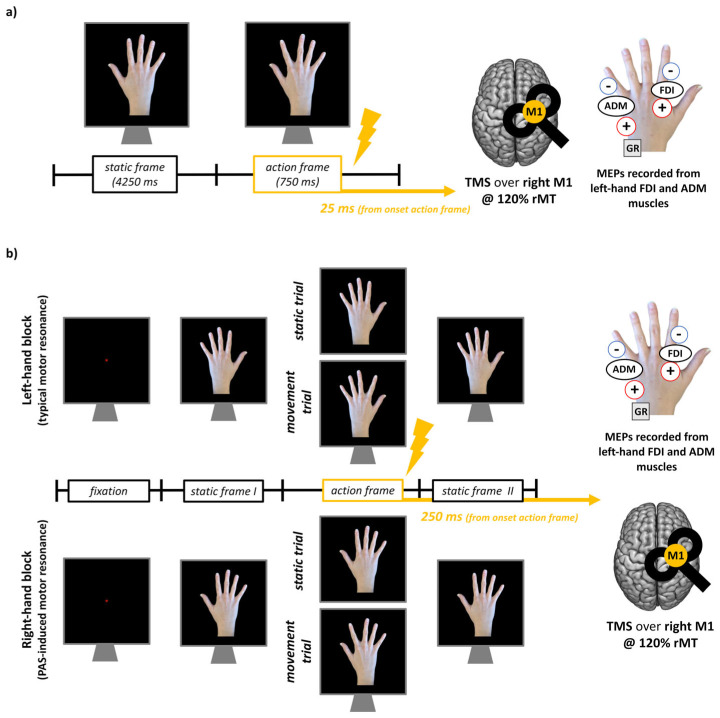
Mirror-paired associative stimulation (m-PAS) protocol (**a**) and action observation task (**b**) used in the datasets considered for the present work. In the action observation tasks, the timing of the frames slightly varied according to the study from where motor-evoked potentials (MEP) data were taken (see: [12,15,16] for further details). m-PAS parameters were the same in every study.

The m-PAS is a visuomotor version of the PAS protocol, where a TMS pulse over the right M1 is repeatedly coupled with a moving right hand (hence ipsilateral to stimulation) to promote the induction of atypical motor resonance responses [12,29]. The m-PAS consisted of 180 paired stimulations delivered at a rate of 0.2 Hz (total duration: 15 min). Each trial started with a frame depicting a right hand in an egocentric perspective at rest. After 4250 ms, a second frame depicting the same hand performing an abduction movement with the index finger appeared for 750 ms, giving rise to apparent motion. We delivered a TMS pulse over the right M1 at a 120% individual resting motor threshold (rMT) after 25 ms from the onset of this second frame (Figure 1a). The correct timing of the frames was verified using a photodiode.

In all our datasets, we assessed motor resonance before and after m-PAS administration during a standard passive action observation task where participants had to observe, in separate blocks, a left or a right hand performing index finger abduction movements [12,15,16]. As for the m-PAS, the rapid succession of two frames—one depicting the hand at rest (static frame) and the other depicting it performing the movement (action frame)—gave the illusion of apparent motion. Regardless of the block (i.e., depicting left or right hands), TMS was always delivered over the right M1 at 120% rMT. During static trials, TMS was delivered while participants observed the hand at rest. During movement trials, TMS was delivered 250 ms from the onset of the action frame, depicting the abduction movements of the index finger (Figure 1b). Half of the trials in each block depicted static hands and half moving ones. We instructed participants to keep their hands relaxed and out of view and carefully observe these visual stimuli and, as an attentive task similar to the control where participants were looking at the computer screen, to respond when a red dot appeared on the depicted hand (10% of total task trial, which we excluded from following analysis). Trial duration and the total number of stimuli in each block slightly varied across studies, and we referred the reader to the original works for more details [12,15,16]; each block lasted about 5 min.

m-PAS and action observation tasks were under computer control, running on E-Prime software (E-Prime 2.0, Psychology Software Tool, Inc., Sharpsburg, PA, USA).

### 2.3. TMS

We delivered TMS pulses with a biphasic figure-of-eight coil (diameter = 70 mm) connected to a Magstim Rapid 2 (Magstim, Whitland, UK) or a Nexstim Eximia stimulator (Nexstim, Helsinki, Finland). We found the motor hotspot of the left FDI muscle by moving the coil in 5 mm steps around the presumed right hemisphere motor hand area by using a slightly supra-threshold stimulus and recording MEPs. Based on the specific experiment, the individual rMT was determined either as the lowest TMS intensity (expressed as a percentage of the maximum stimulator output) that could evoke an MEP of at least 50 µV in the FDI muscle in 5 out of 10 trials [36] or using the parameter estimation by the sequential testing method [37]. On average, the mean rMT of our sample was (mean ± SD) 44.4 ± 10.8% (with no statistically significant difference concerning the procedure adopted, *t* = 0.231, *p* > 0.673). The stable TMS coil placement and position were constantly monitored during the experimental sessions through neuronavigation software, i.e., SofTaxic Optic 2 (EMS, Bologna, Italy) for data collected with the Magstim Rapid 2 stimulator [12] and the integrated navigated brain stimulation system (Nexstim, Helsinki, Finland) for data collected with the Nexstim Eximia stimulator [15,16]. The coil was consistently positioned tangential to the scalp and angled at 45° to the midline, perpendicular to the targeted hand-knob area. This orientation generated brain currents in the stimulated gyrus in the anterior-to-posterior (first phase)/posterior-to-anterior (second phase) direction.

### 2.4. Electromyographic (EMG) Recording and Preprocessing

In all our datasets, we recorded MEPs from FDI (target muscle, implicated in the index finger movements observed by participants) and ADM muscles (control muscle, not implicated in the movements observed) of the left hand with Signal software (version 3.13, Cambridge Electronic Devices, Cambridge, UK). The EMG signal was sampled at 5000 Hz using a Digitimer D360 amplifier (Digitmer Ltd., Welwyn Garden City, UK) connected to a CED micro1401 A/D converter (Cambridge Electronic Devices, Cambridge, UK). Active electrodes were placed over the muscle bellies; reference ones were placed over the metacarpophalangeal joint of the index and little finger (for FDI and ADM recording, respectively). The ground electrode was positioned over the right ulnar head. The EMG signal was amplified, had a band-pass (10–1000 Hz), notch-filtered, and stored for offline analysis. Data were collected from 100 ms before to 200 ms after the TMS pulse (time window: 300 ms). We analyzed MEPs offline using Signal software (version 3.13). MEP peak-to-peak amplitude was calculated in each trial of the m-PAS and the action observation task between 5 ms and 60 ms from the TMS pulse. Trials presenting artifacts greater than 100 µV in the 100 ms before the TMS pulse and trials in which the MEP amplitude was smaller than 50 µV were excluded from the analysis.

### 2.5. Statistical Analyses

For action observation tasks, we computed a *motor resonance index* [16,38] as the ratio in MEP amplitude between movement and rest trials:

$$\mathrm{motor}\;\mathrm{resonance}\;\mathrm{index}\;(\%)=\frac{\mathrm{MEP}\;\mathrm{amplitude}\;\mathrm{in}\;\mathrm{movement}\;\mathrm{trials}}{\mathrm{MEP}\;\mathrm{amplitude}\;\mathrm{in}\;\mathrm{rest}\;\mathrm{trials}}-1$$

Namely, for every participant and condition, the mean MEP amplitude in trials depicting the movement was divided for MEP amplitude from rest trials of the same condition, which served as a baseline for CSE. The value ‘1’ was subtracted from the ratio to express the percentage relative to the resting condition. As a result, positive values indicated increased CSE due to action observation, reflecting the presence of motor resonance.

MEP data recorded during the m-PAS were normalized using z-point transformation, divided into 6 bins of 30 trials each (i.e., bin 1 = trials 1–30; bin 2 = trials 31–60; bin 3 = trials 61–90; bin 4 = trials 91–120; bin 5 = trials 121–150; and bin 6 = trials 151–180), and the mean peak-to-peak amplitude in each bin was calculated to explore the temporal evolution of CSE during the protocol. Raw MEP values for each muscle are reported in Supplementary Tables S1 and S2 for the action observation task’s conditions and m-PAS bins, respectively.

First, as a quality check to assess and replicate the effect of the m-PAS on motor resonance [12,15,16], we ran a repeated-measures analysis of variance (rmANOVA) on the motor resonance index values with factors ‘viewed Hand’ (left-hand, right-hand), ‘Time’ (pre-PAS, post-PAS), and ‘Muscle’ (FDI, ADM). Then, we assessed whether CSE changed during m-PAS administration with a ‘Bin’ (1, 2, 3, 4, 5, 6) X ‘Muscle’ (FDI, ADM) rmANOVA on normalized MEP amplitude. Finally, given the significant patterns found (see Results), we performed a series of linear regression analyses for each muscle, separated for left- and right-hand observation conditions, aiming to explore whether changing in CSE during the m-PAS predicted the protocol’s aftereffects on typical and atypical motor resonance. Here, we considered the difference between motor resonance index values after and before m-PAS administration (i.e., motor resonance gain) as the dependent variable and the difference between normalized MEP amplitude in the final and first bin of the m-PAS (i.e., m-PAS CSE gain) as the predictor.

All statistical analyses were performed using the software Jamovi 2.6 [39]. Statistical significance was defined as *p* < 0.05. We verified the normality of all variables using the Shapiro–Wilk test and Q-Q plot assessments. For repeated-measures ANOVAs, we used Mauchly’s test to assess data sphericity. When sphericity was violated, the Greenhouse–Geisser correction was applied. Significant effects were further explored with Tukey HSD-corrected post hoc comparisons. We calculated the partial eta-squared (*η_p_*^2^), Cohen’s *d*, and the coefficient of determination (R^2^) in every rmANOVA, *t*-test, and regression, respectively, and reported as effect size (i.e., *η_p_*^2^, *d*) and goodness-of-fit (i.e., R^2^) values. As a rule-of-thumb for interpreting the reported effect size values, *η_p_*^2^ and *d* greater than 0.01/0.06/0.14 and 0.2/0.5/0.8 are considered small/moderate/large effects, respectively [40,41]. R^2^ indicates the percentage of variance in the dependent variable explained by the predictor [42]. The standardized regression coefficient (β) was also reported for linear regressions. Section 3 reports mean ± standard error for each variable.

## 3. Results

### 3.1. Motor Resonance Patterns Before and After m-PAS Administration

Results from the rmANOVA on the motor resonance index to check CSE patterns after m-PAS administration showed a significant ‘viewed Hand’ X ‘Time’ X ‘Muscle’ interaction (*F*_1,80_ = 15.57, *p* < 0.001, *η_p_*^2^ = 0.16), as well as main effect of the factor ‘Muscle’ (*F*_1,80_ = 5.23, *p* = 0.025, *η_p_*^2^ = 0.06) and interaction ‘viewed Hand’ X ‘Time’ (*F*_1,80_ = 21.29, *p* < 0.001, *η_p_*^2^ = 0.21). No other significant effect was found (all *F*s < 2.17, all *p*s > 0.144). As previously performed in all our works [12,15,16], we further explored motor resonance patterns with two separate rmANOVAs, one for each muscle.

**Figure 2:**
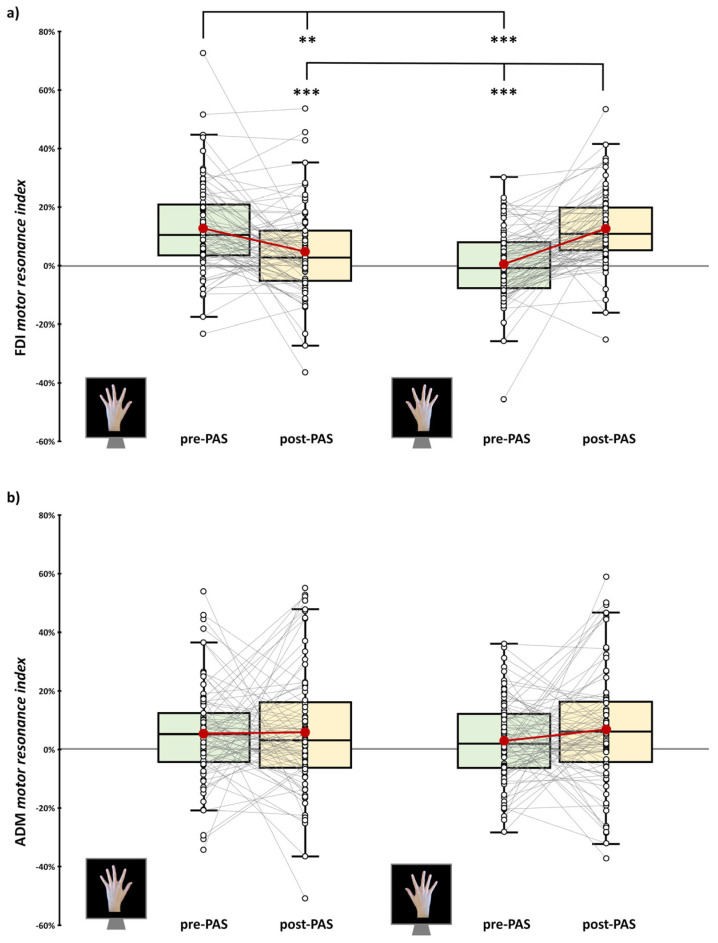
Motor resonance index before (green boxplot) and after (yellow boxplot) m-PAS administration for first dorsal interosseus (FDI, (**a**)) and abductor digiti minimi (ADM, (**b**)) muscles. In the box-and-whisker plots, red dots indicate the means of the distributions. Their median values are reported by the center line. Black-and-white dots show single-subject scores. The box contains the 25th to 75th percentiles of the dataset. Whiskers extend to the largest observation falling within the 1.5 * inter-quartile range from the first/third quartile. Significant Tukey-corrected post hoc comparisons are reported (** = *p* < 0.01; *** = *p* < 0.001).

For the FDI muscle, this analysis showed only a significant main effect of the ‘viewed Hand’ X ‘Time’ interaction (*F*_1,80_ = 52.93, *p* < 0.001, *η_p_*^2^ = 0.4). No other significant effect was found (all *F*s < 0.46, all *p*s > 0.507). Post hoc comparisons showed that, as expected, motor resonance at baseline is present only for left-hand conditions (mean motor resonance index ± standard error: 12.78 ± 1.73%). Motor resonance was not found for right-hand conditions (0.69 ± 1.36%; vs. pre-PAS left-hand motor resonance index: *t*_80_ = 5.57; *p* < 0.001, *d* = 0.62). Following the m-PAS, atypical motor resonance for right-hand movements, conditioned during the PAS, emerged (12.83% ± 1.48%; vs. pre-PAS right-hand motor resonance index: *t*_24_ = 6.63, *p* < 0.001, *d* = 0.74), accompanied with a rewriting of the typical phenomenon. Indeed, after the m-PAS, the motor resonance index for left-hand conditions (4.83 ± 1.16%) was significantly lower than at baseline (*t*_24_ = −3.86, *p* = 0.001, *d* = −0.43) and from the one obtained for right-hand stimuli (*t*_24_ = −4.57, *p* < 0.001, *d* = −0.51; Figure 2a).

For the ADM muscle—not involved in the observed index finger movement, hence acting as a control for the muscle-specificity of motor resonance patterns [12,17]—the rmANOVA showed no significant effects of the factors ‘viewed Hand’ (*F*_1,80_ = 0.14, *p* = 0.714, *η_p_*^2^ < 0.01), ‘Time’ (*F*_1,80_ = 1.03, *p* = 0.314, *η_p_*^2^ = 0.01), and their interaction (*F*_1,80_ = 0.78, *p* = 0.38, *η_p_*^2^ = 0.01) (Figure 2b).

### 3.2. CSE During m-PAS Protocol

**Figure 3:**
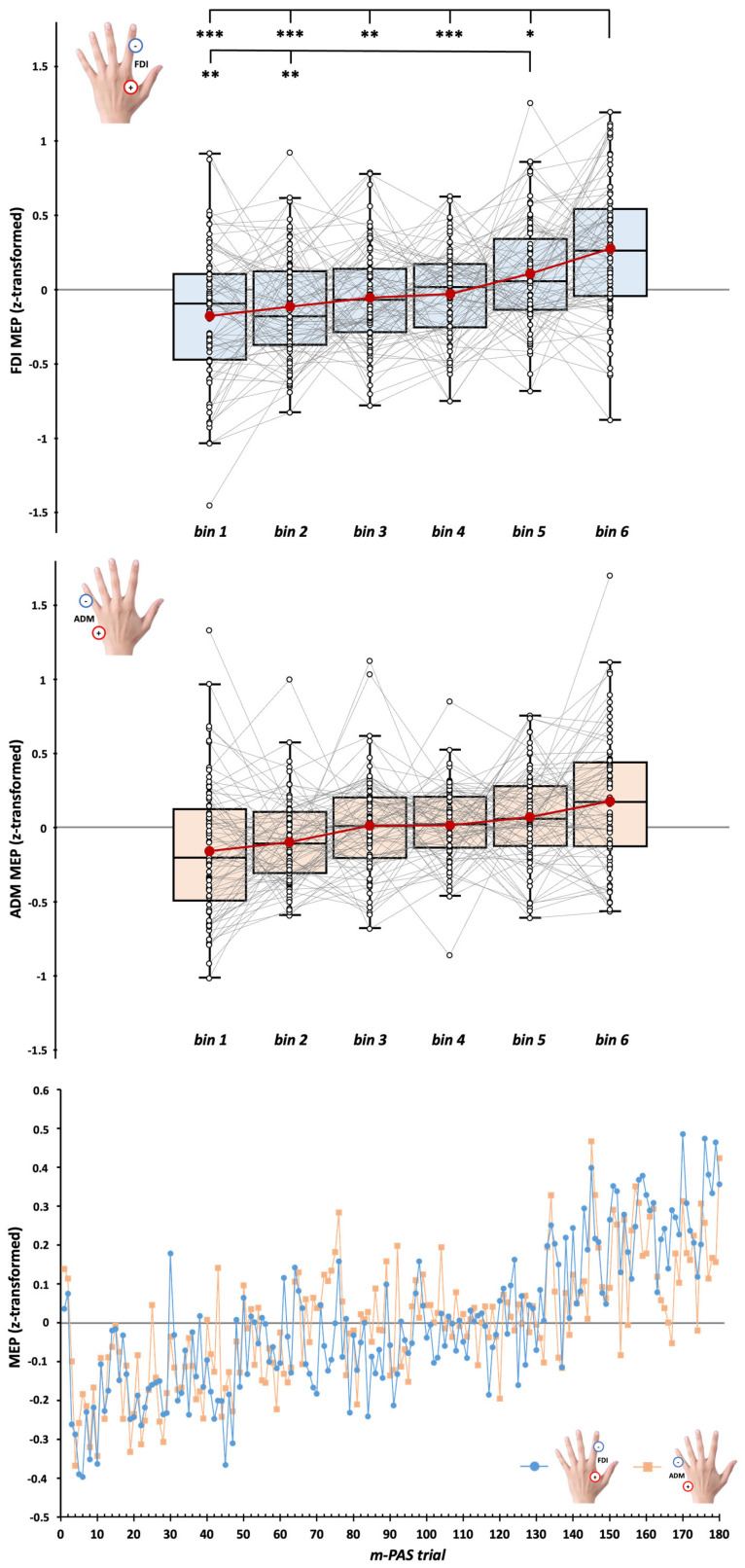
Corticospinal excitability (CSE) temporal profile during the m-PAS for FDI (**upper panel**) and ADM (**middle panel**) muscles according to the 6 bins of 30 trials in which we divided the 180 trials of the m-PAS protocol. (**Lower panel**): mean MEP amplitude at the single-trial level for FDI (blue circles) and ADM (light orange squares). In the box-and-whisker plots, red dots indicate the means of the distributions. Their median values are reported by the center line. Black-and-white dots show single-subject scores. The box contains the 25th to 75th percentiles of the dataset. Whiskers extend to the largest observation falling within the 1.5 * inter-quartile range from the first/third quartile. Significant Tukey-corrected post hoc comparisons for the main effect ‘Bin’ are reported (* = *p* < 0.05; ** = *p* < 0.01; *** = *p* < 0.001).

rmANOVA on (z-transformed) MEPs recorded during the m-PAS showed a significant effect of the main factor ‘Bin’ (*F*_3_._1,255_._3_ = 13.48, *p* < 0.001, *η_p_*^2^ = 0.14) but not of ‘Muscle’ (*F*_1,80_ = 2.83, *p* = 0.096, *η_p_*^2^ = 0.03) or ‘Bin’ X ‘Muscle’ interaction (*F*_3_._5,279_._6_ = 1.78, *p* = 0.142, *η_p_*^2^ = 0.02). Namely, CSE changed during m-PAS administration but with patterns that are not muscle-specific. Post hoc comparisons showed that MEPs recorded during the last 30 trials of the m-PAS protocol (i.e., Bin 6) were significantly higher for all the other bins (vs. Bin 1: *t*_80_ = 5.36; *p* < 0.001, *d* = 0.6; vs. Bin 2: *t*_80_ = 5.08; *p* < 0.001, *d* = 0.56; vs. Bin 3: *t*_80_ = 3.86; *p* = 0.003, *d* = 0.43; vs. Bin 4: *t*_80_ = 4.74; *p* < 0.001, *d* = 0.53; vs. Bin 5: *t*_80_ = 3.01; *p* = 0.039, *d* = 0.34). Furthermore, MEPs in Bin 5 significantly differed from the ones in the first (*t*_80_ = 3.97; *p* = 0.002, *d* = 0.44) and the second bin (*t*_80_ = 3.53; *p* = 0.009, *d* = 0.39). This pattern suggested a progressive increase in CSE during the m-PAS for both FDI and ADM muscles (Figure 3).

### 3.3. CSE Changes During m-PAS and Their Relation with Motor Resonance Modulations

**Figure 4:**
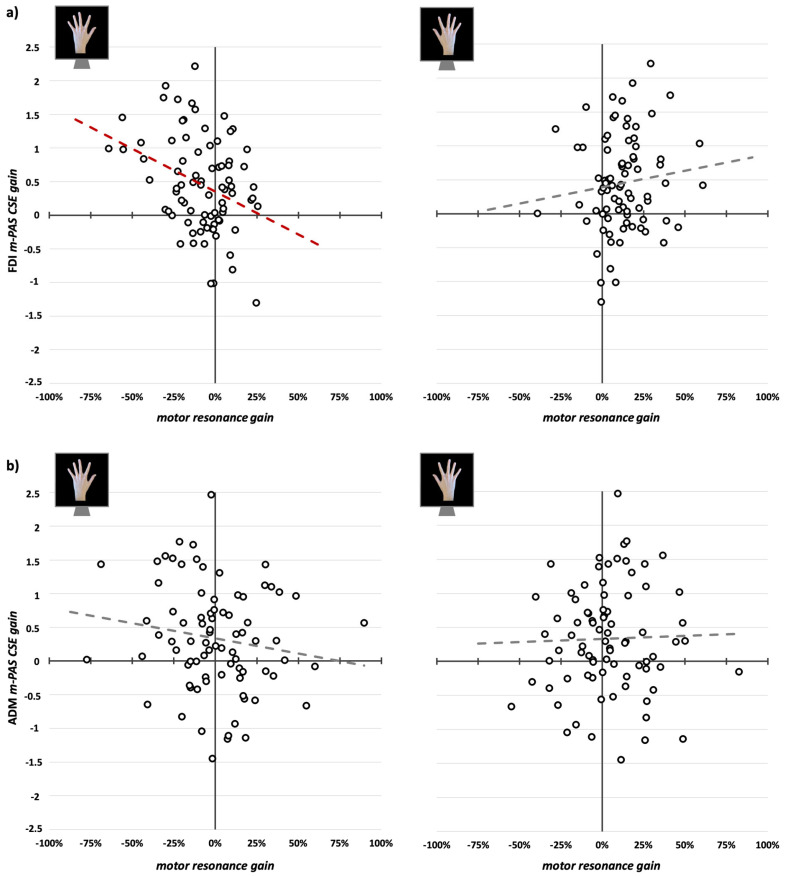
Scatterplots between m-PAS CSE gain (i.e., mean normalized MEP amplitude in m-PAS bin 1 subtracted to the one in bin 6) and motor resonance gain (i.e., motor resonance index before the m-PAS subtracted from values found after its administration) for FDI (**a**) and ADM (**b**) muscles. Dashed lines indicate the linear regression’s fitted line (in red, the significant one found between m-PAS CSE gain and left-hand motor resonance gain for FDI MEPs).

Linear regressions run to explore whether changes in FDI CSE during the m-PAS (m-PAS CSE gain, i.e., mean normalized MEP amplitude in the first bin subtracted from the one in the last bin) predicted the motor resonance gain during the observation of left- or right-hand movements (i.e., motor resonance index before the m-PAS subtracted from values found after its administration) showed a statistically significant relation only for left-hand motor resonance (β = −0.33; *F*_1,79_ = 9.84; *p* = 0.002; R^2^ = 0.11) but not for the right-hand one (β = 0.11; *F*_1,79_ = 1.01; *p* = 0.318; R^2^ = 0.01; Figure 4a). Namely, the difference between the first and last MEP bin recorded during the m-PAS significantly predicted the loss of typical motor resonance (motor resonance gain = −0.08 + −0.087 ∗ m-PAS CSE gain). At variance, the emergence of atypical motor resonance is not associated with CSE modulation during the m-PAS.

Given the significant modulation of CSE during m-PAS found for this muscle, we ran the same regressions for ADM gain indexes. For this muscle, we found that the m-PAS CSE gain did not predict motor resonance gain either during the observation of left- (β = −0.15; *F*_1,79_ = 1.93; *p* = 0.169; R^2^ = 0.02) or right-hand index finger movements (β = 0.03; *F*_1,79_ = 0.06; *p* = 0.803; R^2^ < 0.01; Figure 4b), highlighting the muscle-specificity of the relation previously found.

## 4. Discussion

Our study investigates whether changes in CSE during a visuomotor version of the PAS, the m-PAS [12], predict the protocol’s aftereffects on typical and PAS-induced motor resonance. We found a gradual enhancement of CSE during m-PAS administration, whose magnitude predicts modulation patterns on typical motor resonance (i.e., loss of CSE facilitation during contralateral movement observation) but not on the atypical PAS-conditioned phenomenon (i.e., emergence of CSE facilitation during the observation of ipsilateral movements). Overall, these results provide valuable information on how Hebbian associative plasticity within the AON is built up during the protocol, shedding better light on the possible neurophysiological substrates grounding the m-PAS effectiveness.

### 4.1. Motor Resonance Responses Are Reshaped After the m-PAS

By aggregating data taken from our previous works with the m-PAS [12,15,16], the first novel result is that m-PAS induces not only the emergence of atypical muscle-specific motor resonance for the movement conditioned during the protocol, but also disrupts the typical visuomotor association, reducing the magnitude of motor resonance brought about by observing movements performed with the contralateral hand. That is, the m-PAS not only drives the emergence of motor resonance for actions performed with limbs ipsilateral to the stimulated motor cortex, but it also concurrently inhibits motor resonance for actions performed with contralateral limbs. This pattern was just documented in our more recent work [16]. At the same time, in our previous studies [12,15], only trends for this double aftereffect were seen, likely due to the need for a greater sample size to achieve statistical significance.

As already argued [16], acquiring new motor and visuomotor responses can come at the expense of already established ones, e.g., [8,25,43,44,45,46]. Similarly, following m-PAS administration, a new motor resonance response emerged during the observation of the conditioned movement. However, this atypical reorganization temporarily disrupted pre-existing visuomotor responses within the AON [16]. This evidence points out that the m-PAS, through associative plasticity induction within the AON, transiently induces a complex reorganization of hemispheric-lateralized motor resonance for simple movements, extending beyond the visual stimulus conditioned during the protocol.

### 4.2. Gradual Online Enhancement of CSE During the m-PAS

The second key finding is that CSE is significantly modulated during the m-PAS. Notably, this enhancement is detectable from both FDI and ADM muscles, indicating a general increase in motor cortex excitability during m-PAS. This cortical excitability enhancement occurs specifically during the protocol’s administration. In fact, the analysis of raw MEP amplitude collected during the action observation task did not show any modulation of CSE after m-PAS. Still, m-PAS effects were detected only at the level of motor resonance (see Supplementary Analysis S1). This evidence means that the online CSE enhancement induced by m-PAS dissociates from its offline effects on motor resonance.

The increase in CSE during the m-PAS aligns well with previous studies using cortico-cortical PAS targeting PMv-M1 connectivity and showing a gradual enhancement of M1 excitability during this protocol [32,33], accompanied by the modulation of AON activation proxies after its administration [13,14]. As stated in Section 1, the PMv-M1 pathway is the neurophysiological underpinning binding AON and M1 activations and grounding the motor resonance phenomenon [20]. Besides studies with the cortico-cortical PAS, different works using repetitive and paired-pulse TMS showed that the perturbation of PMv impacts MEP facilitation during action observation, e.g., [21,24,25,26]. Hence, assuming its involvement during the m-PAS is reasonable, even if not directly stimulated [12]. In this framework, the increased CSE during the protocol could be interpreted as evidence that the PMv-M1 pathway is salient for associative plasticity induction during the m-PAS.

An alternative yet complementary hypothesis considers the possible contribution of predictive mechanisms [3,47] during m-PAS administration. Action observation stimuli presented during the m-PAS are highly predictable, considering that participants observed the same visual stimulus of movement always presented every 5 s for 15 min. As the protocol progresses, the AON can likely be engaged in advance, even before the actual observation of the depicted actions. This is corroborated by previous AON studies, with humans and primates showing action anticipation phenomena driven by the presentation of sufficient contextual cues about the movement to be seen, e.g., [48,49,50,51,52]. Furthermore, similar anticipatory mechanisms have been shown to be recruited during a cross-modal version of the PAS, pairing the sight of touch with somatosensory cortical stimulation [10,11]. In this view, the gradual enhancement of CSE could underlie, besides the induction of state-dependent associative plasticity within the motor system likely mediated by the PMv-M1 pathway, an anticipatory M1 engagement by action observation with the protocol progression.

This evidence stresses that the m-PAS acts at a network level [16] rather than influencing a single AON pathway (e.g., cortico-cortical PAS targeting PMv-M1 connectivity [53]). From this perspective, our findings suggest that the gradual enhancement of CSE during m-PAS administration would reflect greater M1 reactivity, with associative plasticity being progressively promoted within the entire AON. If this is true, a dynamic reshaping of AON properties could already occur during the protocol, opening up the chance that CSE modulations detected online could inform the protocol’s aftereffects.

### 4.3. CSE Increase During m-PAS Predicts Only the Rewriting of Typical Motor Resonance

The latter hypothesis is confirmed by the third significant result of our study, namely that the magnitude of CSE increase during the m-PAS predicts muscle-specific aftereffects on typical motor resonance, involving its reduction when viewing actions performed with the contralateral hand. This relation is specific for FDI, i.e., the muscle involved in the observed index finger movement. Hence, even if CSE patterns during the m-PAS are not muscle-specific, reflecting a gradual enhancement of motor system excitability unrelated to the features of the conditioned visual stimulus, their relation with motor resonance modulations follows the typical muscle-specific feature of this phenomenon [17,19]. On the contrary, the emergence of the experimentally induced novel motor resonance effect is unrelated to the online CSE enhancement.

Altogether, these results (i.e., online CSE change predicts the disruption of ‘in place’ visuomotor associations but not the building up of new ones) suggest that the rewriting of typical motor resonance already occurs during the m-PAS in the targeted motor system, while the induction of new visuomotor association likely took place later, i.e., once the protocol ended. It follows that the CSE enhancement is a successful predictor of some but not all the complex modulations of AON activity induced by the m-PAS, likely reflecting Hebbian associative plasticity induction. In this regard, TMS-induced plasticity could also take place in the unstimulated M1. For instance, different clinical studies showed a functional reorganization of inter-hemispheric M1-M1 communication following rehabilitative training based on action observation treatments or mirror therapies, e.g., [54,55,56,57,58]. Thus, it is reasonable to speculate the bilateral recruitment of the AON by the m-PAS, resulting in modulation of the contralateral (here the left) M1 excitability that might explain the protocol’s aftereffects on the experimentally induced motor resonance.

### 4.4. Limitations and Future Directions

The present work has some limitations that need to be highlighted. Firstly, we did not have a control condition that could disambiguate if the online CSE enhancement is specific for this version of the m-PAS. Future studies could investigate whether alternative or ineffective m-PAS protocols lead to similar online CSE enhancement. Secondly, our speculations on the neurophysiological underpinning of this enhancement are based on a peripheral measure of cortical reactivity (i.e., MEPs). Future works can likely integrate neuroimaging techniques (e.g., electroencephalography, functional magnetic resonance imaging) during m-PAS administration to better track the cortical signatures of associative plasticity induction.

All this considered, the results of the present work open up to intriguing and thought-provoking future directions. For instance, studies could introduce hybrid, cortico-cortical versions of the m-PAS where single-pulse TMS over M1 is replaced with paired-pulse targeting specific AON nodes to disentangle better the contribution of specific pathways in the effects found on CSE and motor resonance during and after m-PAS. At the same time, the contribution of specific AON nodes and pathways could be further explored by varying the features of the visual stimulus of movement conditioned, e.g., replacing the intransitive, simple movement with more ecological and goal-directed actions, which are known to recruit the AON to a greater extent [59,60]. Similarly, less foreseeable versions of the m-PAS could be tested, assessing the influence of the protocol’s predictability on online and offline markers of associative plasticity induction.

These findings suggest the potential for adapting m-PAS protocols for clinical use, with parameters customized to each patient’s clinical profile. For example, the protocol could include the observation of specific movements or gestures requiring rehabilitation, fostering adaptive plasticity in the motor system through AON recruitment. Additionally, m-PAS parameters—such as protocol duration, the number of visual stimuli, and their frequency—could be optimized to match the patient’s attentional capacity. In this vein, a recent proof-of-principle study on stroke survivors suggests that PAS protocols acting on visuomotor mirroring can induce associative plasticity within a damaged motor system [61].

## 5. Conclusions

In conclusion, our findings corroborate the evidence that m-PAS effects on motor resonance responses are the product of a network-wide reorganization of AON functioning, impacting a vast range of cortical regions with modulations at the CSE level already trackable during the protocol administration. Crucially, the online CSE enhancement can be used to predict just a part of m-PAS aftereffects, particularly solely modulations more strictly related to the typical, hemispheric-specific motor cortex recruitment during action observation. This evidence aligns well with the results of our recent work integrating m-PAS and neuroimaging, which showed a complex, brain-wide, and frequency-specific reorganization of right M1 functional connectivity during action observation after m-PAS administration, suggesting that typical and experimentally induced motor resonance are not superimposable phenomena, relying on distinct oscillatory dynamics and connectivity patterns [16].

## Data Availability

The dataset and analyses of the present study are publicly available on Open Science Framework (OSF): https://osf.io/7gsut/ (accessed on 25 February 2025).

## Supplementary Materials

The following supporting information can be downloaded at: https://www.mdpi.com/article/10.3390/brainsci15030257/s1, Supplementary Table S1. Raw MEP amplitude (mean ± standard error) for FDI and ADM muscles in the four trial typologies of the action observation task before and after m-PAS administration. Supplementary Table S2. Raw MEP amplitude (mean ± standard error) for FDI and ADM muscles in the 6 bins in which we divided the 180 trials of the m-PAS. Supplementary Analysis S1. rmANOVA on raw MEP amplitude recorded during the action observation tasks.

## Abbreviations

The following abbreviations are used in this manuscript:

ADM	Abductor digiti minimi
AON	Action observation network
CSE	Corticospinal excitability
FDI	First dorsal interosseus
m-PAS	Mirror-paired associative stimulation
M1	Primary motor cortex
MEP	Motor-evoked potential
PAS	Paired associative stimulation
PMv	Ventral premotor cortex
rmANOVA	Repeated measures analysis of variance
rMT	Resting motor threshold
SD	Standard deviation
TMS	Transcranial magnetic stimulation
