# Supplementary material for: Tracking Changes in Corticospinal Excitability During Visuomotor Paired Associative Stimulation to Predict Motor Resonance Rewriting
Source: Brain Sci. 2025 Feb 27;15(3):257. doi: 10.3390/brainsci15030257 (PMC11940033; doi:10.3390/brainsci15030257)
Supplement: Supplementary file 1 [file brainsci-15-00257-s001.zip › brainsci-3509146-supplementary.pdf]

Tracking changes in corticospinal excitability during visuomotor paired associative stimulation to predict motor resonance rewriting

Giacomo Guidali & Nadia Bolognini

- SUPPLEMENTARY MATERIALS -

Muscle	Trial	pre-PAS (mV)	post-PAS (mV)
FDI	left-hand static	1.63 ± .11	1.74 ± .12
	left-hand movement	1.81 ± .12	1.79 ± .12
	right-hand static	1.82 ± .11	1.57 ± .09
	right-hand movement	1.81 ± .11	1.77 ± .1
ADM	left-hand static	1.04 ± .08	1.05 ± .1
	left-hand movement	1.09 ± .09	1.07 ± .09
	right-hand static	1.02 ± .08	1.04 ± .1
	right-hand movement	1.04 ± .1	1.09 ± .1

Supplemental Table S1. MEP amplitude raw data (mean ± standard error) from FDI and ADM muscles in the four trial typologies of the action observation task, before and after m-PAS administration.

Muscle	Bin 1 (mV)	Bin 2 (mV)	Bin 3 (mV)	Bin 4 (mV)	Bin 5 (mV)	Bin 6 (mV)
FDI	1.7 ± .1	1.79 ± .12	1.86 ± .12	1.88 ± .12	1.98 ± .12	2.14 ± .13
ADM	1.09 ± .09	1.14 ± .09	1.19 ± .1	1.2 ± .1	1.23 ± .1	1.31 ± .11

Supplemental Table S2. MEP amplitude raw data (mean ± standard error) from FDI and ADM muscles in the 6 bins in which we divided the 180 trials of the m-PAS.

Supplemental Analysis S1

To explore whether the CSE enhancement found during the m-PAS, reflecting a general increasing of M1 excitability, is persistent also after protocol's administration, we run a 'Trial type' (static, movement) X 'viewed Hand' (left-hand, right-hand) X 'Time' (pre-PAS, post-PAS) X 'Muscle' (FDI, ADM) on MEP amplitude recorded during the action observation task and from which we derived our *motor resonance*

indexes. Besides the significant quadruple interaction ($F_{1,80}$ = 23.32, p < .001, η_p^2 = .23), main factor 'Time' ($F_{1,80}$ = .17, p = .685, η_p^2 < .01), as well as interactions 'Time X Muscle' ($F_{1,80}$ = 1.73, p = .192, η_p^2 = .02), 'Time X viewed Hand' ($F_{1,80}$ = 1.29, p = .259, η_p^2 = .02), 'Time X Trial type' ($F_{1,80}$ = 2.42, p = .124, η_p^2 = .03), and 'Time X Trial type X Muscle' ($F_{1,80}$ = 2.34, p = .13, η_p^2 = .03) were not statistically significant. This pattern of results suggests that CSE modulation (and hence M1 excitability) were specific for the viewed hand, as already highlighted by the analyses reported in the main text. Crucially, CSE is not overall modulated before and after m-PAS administration.
